# LncRNA *GATA3-AS1*-mediated m*iR-2116-5p* interaction modulates *TCF12* and contributes to podocyte dysfunction in childhood nephrotic syndrome

**DOI:** 10.1016/j.clinsp.2026.101053

**Published:** 2026-07-16

**Authors:** Xin Tong, Yong Zhao, Peng Huang

**Affiliations:** a Department of Nephrology, Dazhou Central Hospital, Dazhou, China; b Jiangxi Beizheng Stell Cell Biotechnology Co., Ltd, Jiangxi, China; c Department of Nephrology, The Affiliated Hospital of Youjiang Medical University for Nationalities, Baise, China; d Key Laboratory of Medical Research Basic Guarantee for Immune-Related Diseases Research of Guangxi, Baise, China

**Keywords:** Childhood nephrotic syndrome, LncRNA *GATA3-AS1*, miR-2116-–5p, *TCF12*, Podocyte

## Abstract

**Objectives:**

Childhood Nephrotic Syndrome (NS) is a common kidney disease. This study investigates the role of LncRNA *GATA3-AS1* and miR-2116–5p in childhood NS and their impact on podocyte function.

**Methods:**

This study employed qRT-PCR to detect the levels of *GATA3-AS1* and miR-2116–5p in serum and evaluated their diagnostic value. An in vitro injury model was established by treating MPC5 podocytes with TGF-β1. Following the overexpression of *GATA3-AS1*, miR-2116–5p, or *TCF12*, cell proliferation, inflammatory responses, and oxidative stress were assessed. RNA interactions were examined using dual-luciferase reporter and RIP assays, and podocyte-specific proteins were analyzed by Western Blot.

**Results:**

In childhood NS patients, serum *GATA3-AS1* is downregulated and miR-2116–5p upregulated, with a negative correlation. ROC curves show they have good diagnostic value. In vitro, *GATA3-AS1* overexpression restored TGF-β1 -inhibited cell proliferation, reduced inflammatory factors, lowered MDA, and raised SOD activity, with miR-2116–5p mimic reversing this effect. Bioinformatics analysis revealed *TCF12* as a high-confidence target gene of miR-2116–5p, and luciferase assays confirmed the binding between miR-2116–5p and *TCF12*. Childhood NS patients have lower serum *TCF12* than controls, and TCF12 overexpression can repair miR-2116–5p -induced podocyte damage. TCF12 overexpression can restore the expression of podocyte marker proteins such as Synaptopodin and Podocin, and inhibit Cleaved Caspase-3-mediated apoptosis.

**Conclusions:**

LncRNA *GATA3-AS1* is downregulated in pediatric NS and mediates the expression of miR-2116–5p, thereby influencing *TCF12*, a mechanism that may potentially have a protective effect on the kidneys in children.

## Highlights

• Serum LncRNA *GATA3‑AS1* is downregulated and *miR‑2116‑5p* upregulated in children with NS.
• *GATA3‑AS1* acts as a ceRNA for *miR‑2116‑5p*, and their levels show diagnostic potential.
• Overexpression of *GATA3‑AS1* alleviates TGF‑β1‑induced podocyte injury.
• *miR‑2116‑5p* directly targets TCF12 and reverses *GATA3‑AS1*‑mediated protection.
• The *GATA3‑AS1*/*miR‑2116‑5p*/*TCF12* axis regulates podocyte marker expression and apoptosis.

## Introduction

Childhood Nephrotic Syndrome (NS) is a common kidney disease characterized by massive proteinuria, hypoalbuminemia, hyperlipidemia, and edema, with high morbidity and mortality rates. NS is typically diagnosed in children aged 1- to 6-years, initially presenting as facial swelling that may gradually spread throughout the body.^1^ NS can be classified into primary and secondary types, among which primary NS is mostly caused by glomerular diseases, while secondary NS may be triggered by various systemic diseases or infections.^2^ It is currently believed that the pathogenesis of NS is associated with various factors such as immune response, alterations in renal hemodynamics, and endocrine disorders.^3^ The pathological process is complex and can lead to a gradual deterioration of renal function, potentially progressing to chronic renal failure.^4^ Currently, the diagnosis of NS relies on clinical manifestations, laboratory tests, and renal biopsy, but traditional markers cannot reflect its damage at an early stage.^5^ Although glucocorticoids and immunosuppressants are commonly used in treatment, their efficacy is limited with numerous side effects, and they are not effective for refractory NS.^6^ Therefore, in-depth research into the pathogenesis of childhood NS and the identification of novel early diagnostic markers and therapeutic targets are crucial for improving the clinical outcomes of childhood NS.

The critical roles of non-coding RNAs (such as lncRNA and miRNA) in various diseases have gradually garnered attention.^7^ miRNA influences key processes such as cell proliferation, differentiation, apoptosis, and immune responses by regulating multiple disease-related genes.^8^ lncRNA, as a competitive endogenous RNA (ceRNA), sponges miRNAs to interfere with their repression of target genes, thereby becoming a crucial node in the gene regulatory network.^9^ *GATA3-AS1* is an antisense transcript located at the GATA3 locus, extensively involved in various physiological and pathological processes.^10^ In breast cancer, GATA3 gene mutations can impair its DNA-binding activity and protein stability, alter gene regulatory networks, and promote tumor invasion and growth.^11^ In preeclampsia, LncRNA *GATA3-AS1* promotes trophoblast cell apoptosis and inhibits their proliferation, migration, and invasion capabilities by acting as a molecular sponge for miR-488–3p and regulating ROCK1 expression.^12^ Research has found that GATA3 is a key transcription factor in embryonic kidney development. GATA3 is specifically expressed in the glomerulus, playing a crucial role in maintaining its function and preventing glomerular diseases,^13^ offering protective effects on the glomerulus.^14^ In addition, relevant reports have indicated that the downregulation of *GATA3-AS1* is associated with the aggressive phenotype of renal cell tumors.^15^ However, its expression pattern and functional significance in childhood NS have not yet been explored. Therefore, this study hypothesizes that *GATA3-AS1* may play a potential role in NS.

In the initial phase of this investigation, the lncRNASNP2 database was employed to screen for miRNAs with potential interactions against *GATA3-AS1*. This predictive analysis identified miR-2116–5p as a principal candidate, a designation based on its high binding affinity score derived from the computational model. Further literature review revealed that research on this molecule in the field of pediatric NS and even kidney diseases remains a blank, which presents an opportunity for exploring novel regulatory mechanisms. Although not reported in NS, recent evidence suggests its association with microvascular complications. miR-2116–5p is upregulated in the serum of patients with Diabetic Retinopathy (DR), a disease also characterized by microvascular damage and capable of promoting retinal endothelial cell dysfunction.^16^^,^^17^ Given the common pathological mechanisms of endothelial/podocyte injury in diabetic microvascular complications and the similarities between retinal and glomerular vascular lesions.^18^ Based on this, we propose the hypothesis that *GATA3-AS1* may influence the development of childhood NS by specifically adsorbing miR-2116–5p, thereby affecting podocyte function. Preliminary studies suggest that *GATA3-AS1* may play a role in renal pathology.^19^ However, its specific function and molecular mechanisms in children with NS remain unclear. An important paradigm of LncRNA function is acting as a competitive endogenous RNA, which specifically sequesters microRNAs through sequence complementarity, thereby relieving miRNA-mediated repression of their target genes.^20^ Therefore, by integrating computational predictions, the established ceRNA paradigm, and the potential renoprotective role of *GATA3-AS1*, this study hypothesizes that in children with NS, *GATA3-AS1* may participate in the disease pathology by acting as a ceRNA to adsorb miR-2116–5p, thereby regulating the expression of downstream target genes.

Therefore, based on the aforementioned reports, this study aims to delve into the mechanism of lncRNA *GATA3-AS1* in childhood NS, particularly its molecular mechanism in regulating podocyte function through the miR-2116–5p-mRNA axis. Aimed at discovering new early diagnostic markers and therapeutic targets, it is expected to provide stronger support for the clinical management of childhood NS.

## Materials and methods

### Research subjects

This study enrolled 128 healthy individuals and 105 childhood NS patients. All participants were recruited from Dazhou Central Hospital between January 2022 and December 2023. The study subjects are children under 10-years of age. Children in the NS group must meet the following diagnostic criteria: 24-hour urinary protein excretion > 50 mg/kg, plasma albumin < 30 g/L, elevated blood lipids (cholesterol > 5.7 mmoL/L or triglycerides > 1.7 mmoL/L), edema (in the lower limbs or throughout the body). The healthy control group must have no history of kidney disease, no proteinuria or hematuria, and normal renal function. This study was performed in line with the principles of the Declaration of Helsinki. Approval was granted by the Ethics Committee of Dazhou Central Hospital (approval number: 2021–05–14). The legal guardians of all study participants are required to sign the informed consent form. Exclusion criteria included: concurrent systemic diseases, use of immunosuppressants or glucocorticoids within the past three months that may affect renal function, presence of hereditary or congenital kidney diseases, being in the acute phase of infection, or having secondary childhood NS.

### Quantitative real-time PCR analysis

Additive-free serum tubes were used to collect blood samples, which were left to stand at room temperature for 30‒60 min to allow blood coagulation. The serum was then separated by centrifugation at 1900 g and 4 °C for 15-minutes. The upper serum was then gently aspirated and transferred into sterile cryovials, and stored in a −80 °C freezer for subsequent RNA extraction. The relative expression levels of miR-2116–5p and LncRNA *GATA3-AS1* in serum and cells were detected using qRT-PCR. Total RNA was extracted using the miRNeasy Serum/Plasma Kit (Qiagen), then reverse-transcribed into cDNA using the PrimeScript RT Kit (TaKaRa), followed by qRT-PCR performed with SYBR Premix Ex Taq (TaKaRa). GAPDH was used for the normalization of LncRNA *GATA3-AS1*, and U6 was used for the normalization of miR-2116–5p, with relative expression levels calculated using the 2^-ΔΔCt^ method.

### Cell culture and induction

The mouse renal podocyte cell line MPC5 cells (Purchased from ATCC, USA) were grown in high-glucose DMEM with 10% FBS and 1% penicillin-streptomycin, incubated at 37 °C with 5% CO_2_. Thaw the cryopreserved MPC5 cells rapidly in a water bath, transfer them to a centrifuge tube with complete medium, and collect the cells by centrifugation at 1000 rpm for 5 min. After resuspension in fresh complete medium, inoculate into culture flasks. When the cells reach 80% confluence, digest them with 0.25% trypsin-EDTA (Gibco) and continue culturing at a 1:3 split ratio. For TGF-β1 induction, MPC5 cells were seeded in 6-well plates. After culturing until 80% confluence, cells in the TGF-β1 group were exposed to a 10 ng/mL TGF-β1 solution. The control group was supplemented with an equal volume of serum-free medium and continued to be cultured for 24-hours.

### Cell transfection

Perform cell transfection on the TGF-β1-induced MPC5 cells mentioned above. When transfecting the *GATA3-AS1* overexpression plasmid, follow the instructions provided with Lipofectamine 3000 (Invitrogen). 2 μg of plasmid and 10 μL of Lipofectamine 3000 were diluted separately in serum-free medium, incubated at room temperature for 5 min, mixed, and incubated for an additional 15 min. By gently shaking the mixture to evenly distribute the plasmid within the cells, subsequent experiments were conducted 48-hours after transfection. For the group co-transfected with miR-2116–5p mimic, the transfection procedure was the same but included 50 nM miR-2116–5p mimic alongside the overexpression plasmid. For the rescue experiment, we transfected a plasmid designed for overexpressing full-length human TCF12 (designated oe-TCF12) into cells, and the observed restoration of TCF12 levels is attributable to the transfection of this overexpression construct.

### Cell proliferation

Cell proliferation was assessed via CCK-8 method. MPC5 cells were seeded in a 96-well plate at 100 μL/well (≈5 × 10^3^ cells). Set up three replicates to ensure data reliability. At 0, 24, 48, and 72 h, 10 μL CCK-8 solution (Dojindo Molecular Technologies, Inc.) was added per well, followed by 4-hour incubation at 37 °C with 5% CO_2_. OD values at 450 nm were measured using a microplate reader.

### Inflammatory factor detection

Collect the cell supernatant and perform the operation according to the instructions of the ELISA kit (R&D Systems). Add the standard and samples to the wells pre-coated with specific antibodies, incubate and wash, then add enzyme-labeled antibodies, incubate again, and finally add the substrate solution for color development. Finally, the absorbance is measured at a specific wavelength using a microplate reader, and the concentrations of various inflammatory factors in the samples are calculated through the standard curve.

### Detection of oxidative stress markers

The intracellular MDA content and SOD activity were detected using the colorimetric method. The MDA content was determined by the thiobarbituric acid reaction method using the MDA assay kit (Cayman Chemical), and the absorbance was measured at a wavelength of 530 nm. The SOD activity was determined by the xanthine oxidase method using the SOD assay kit (Cayman Chemical), and the absorbance was measured at a wavelength of 450 nm. The SOD activity was calculated according to the formula provided in the kit.

### Western blot analysis

After extracting total cellular proteins, the concentration was determined using the BCA method. Equal amounts of proteins were separated by SDS-PAGE electrophoresis, transferred to PVDF membranes, and blocked with 5% skim milk. Then incubate with the primary antibody at 4 °C overnight. Anti-Synaptopodin antibody, anti-Podocin antibody, anti-Cleaved Caspase-3 antibody, and anti-GAPDH antibody were all purchased from Cell Signaling Technology company, with a dilution of 1:1000. After washing with TBST, incubate with the corresponding secondary antibody at room temperature for 1 hour. Develop using ECL chemiluminescence reagent, and analyze the band gray values with ImageJ software. The expression of target proteins was normalized to GAPDH as an internal reference.

### Dual-luciferase assay validation

First, the LncRNA *GATA3-AS1* fragment containing the miR-2116–5p binding site was cloned into the psiCHECK-2 vector to construct the WT plasmid, and point mutations were introduced at the binding site to construct the MUT plasmid. Then, cells were seeded in 24 ‒ well plates at 5 × 10^4^ cells per well. When they reached 70% confluence, WT or MUT plasmids (0.5 μg/well) were co-transfected with miR-2116–5p mimic or mimic NC into cells using Lipofectamine 3000, and three replicates were set for each experimental group. Forty-eight hours post-transfection, luciferase activity was measured to determine the direct binding between miR ‒ 2116 ‒ 5p and *TCF12*.

### RNA immunoprecipitation (RIP)

EZ-Magna RIP™ RNA-Binding Protein Immunoprecipitation Kit (Millipore) was applied for RIP. Immunoprecipitation was performed on TGF-β1-treated MPC5 cell lysates using anti-Ago2 antibody (or isotype control IgG), followed by extraction of co-precipitated RNA. The enrichment levels of *GATA3-AS1* and miR-2116–5p were respectively detected by qRT-PCR.

### Bioinformatics

Predict the potential target genes of miR-2116–5p using three databases: miRmap (https://mirmap.ezlab.org/), miRWalk (http://mirwalk.umm.uni-heidelberg.de/), and TargetScan (https://www.targetscan.org/vert_80/). Through Venn analysis, identify the commonly predicted target genes to enhance the reliability of the prediction. Subsequently, GO enrichment (https://www.geneontology.org) and KEGG pathway (https://www.genome.jp/kegg/) analyses were performed on the predicted common target genes. Finally, the miRNA-target gene network was constructed using the STRING (https://cn.string-db.org/) database.

### Data analysis

Data were analyzed with SPSS 26.0 and GraphPad Prism. They are shown as mean ± SD. Group differences were assessed via independent ‒ samples *t*-test or ANOVA. Correlations were determined using Pearson's test. All in vitro experiments in this study were conducted with three independent biological replicates. Within each biological replicate, we performed three technical replicate measurements to ensure data accuracy. A p-value < 0.05 was considered statistically significant.

## Results

### Basic characteristics

The baseline comparison between childhood NS patients and the healthy group (Table 1) revealed that the levels of WBC, PLT, BUN, serum creatinine, and PRO in the NS group were significantly higher than those in the healthy control group (all p-values < 0.001). However, there were no significant differences between the two groups in terms of age, BMI, and gender ratio (all p-values > 0.05).

**Table 1:** Baseline analysis between the childhood nephrotic syndrome group and the healthy group.

Variable	Controls (n = 128)	Childhood NS (n = 105)	*p*-value
Age (year)	3.27±1.76	3.51±1.77	0.302
BMI (kg/m^2^)	24.11±3.14	24.34±4.04	0.616
Gender (M/F)	65/63	61/44	0.267
WBC (10^9^/L)	6.11±2.32	11.67±2.07	<0.001
PLT (10^9^/L)	171.24±5.70	352.43±10.99	<0.001
BUN (mmol/L)	3.12±0.31	7.35±2.01	<0.001
Serum creatinine (μmol/L)	26.86±4.35	64.76±8.55	<0.001
PRO (mg/24 h)	116.95±7.78	152.53±9.87	<0.001

Note: BMI, body mass index; WBC, white blood cell count; PLT, platelet count; BUN, blood urea nitrogen; PRO, proteinuria; M/F, male/female. The date was presented as mean ± SD.

### The expression and correlation of LncRNA GATA3-AS1 and miR-2116–5p in childhood NS

**Fig. 1:**
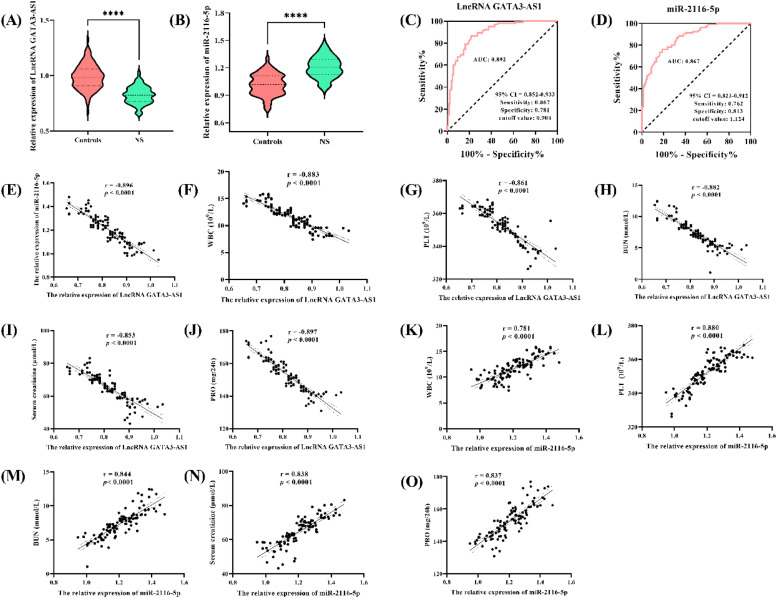
Expression and correlation analysis of LncRNA *GATA3-AS1* and miR-2116–5p in childhood NS. Relative levels of *GATA3-AS1* (A) and miR-2116–5p (B) in NS patients versus healthy controls; ROC curves of *GATA3-AS1* (C) and miR-2116–5p (D); Correlation of *GATA3-AS1* with miR-2116–5p (E) and clinical indicators WBC (F), PLT (G), BUN (H), serum creatinine (I), PRO (J); Correlation of miR-2116–5p with WBC (K), PLT (L), BUN (M), serum creatinine (N), PRO (O). Continuous variables were compared by independent samples *t*-test, categorical variables by Chi-square test. ****p < 0.0001.

In the childhood NS group, the relative level of *GATA3-AS1* (0.82 ± 0.08) was significantly lower than that in the healthy control group (0.99 ± 0.11, p < 0.0001, Fig. 1A), while the relative level of miR-2116–5p (1.21 ± 0.12) was significantly higher than that in the healthy control group (1.02 ± 0.12, p < 0.0001, Fig. 1B). ROC revealed that the AUC of *GATA3-AS1* was 0.892 (95% CI 0.852‒0.933, Fig. 1C), with a sensitivity of 0.867, specificity of 0.781, and a cutoff value of 0.904. The AUC of miR-2116–5p is 0.867 (95% CI 0.823‒0.912, Fig. 1D), with a sensitivity of 0.762, a specificity of 0.813, and a cutoff value of 1.124, both of which possess certain diagnostic value. Correlation analysis showed that GATA3 ‒ AS1 expression was negatively correlated with miR-2116–5p (Fig. 1E), WBC (Fig. 1F), PLT (Fig. 1G), BUN (Fig. 1H), serum creatinine (Fig. 1I), and PRO (Fig. 1J), all p < 0.0001. In contrast, the expression of miR-2116–5p showed a significant positive correlation with WBC (Fig. 1K), PLT (Fig. 1L), BUN (Fig. 1M), Serum creatinine (Fig. 1N), and PRO (Fig. 1O), all p < 0.0001.

### The impact of LncRNA GATA3-AS1 on podocyte function and its mechanism

**Fig. 2:**
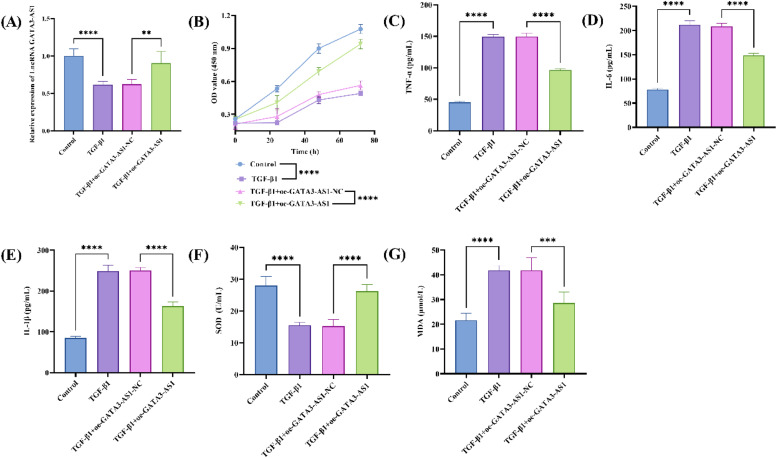
Impact of LncRNA *GATA3-AS1* on TGF-β1-induced MPC5 cell function and mechanism analysis. The relative expression of *GATA3-AS1* in TGF-β1-induced MPC5 cells (A); The effects of LncRNA *GATA3-AS1* overexpression on TGF-β1-induced MPC5 cell proliferation capacity (B), TNF-α (C), IL-6 (D), IL-1β (E) secretion, SOD activity (F), and MDA content (G); n = three biological replicates; Data were analyzed using one-way ANOVA for intergroup comparisons, followed by Tukey's post hoc test; ****p < 0.0001, ***p < 0.001, **p < 0.01.

Further analysis of the results revealed that, compared to the Control, the relative level of LncRNA *GATA3-AS1* was significantly reduced in TGF-β1-treated samples, and TGF-β1 could markedly inhibit the expression of *GATA3-AS1* (Fig. 2A, p < 0.0001). The CCK-8 assay results demonstrated that TGF-β1 treatment significantly inhibited the proliferative capacity of MPC5 cells, while overexpression of *GATA3-AS1* markedly restored cell proliferation (Fig. 2B, p < 0.0001). The ELISA results showed that TGF-β1 treatment significantly increased the secretion levels of TNF-α (Fig. 2C), IL-6 (Fig. 2D), and IL-1β (Fig. 2E), while overexpression of *GATA3-AS1* markedly reduced the secretion of these inflammatory factors. In addition, TGF-β1 treatment reduced SOD activity and increased MDA content, while overexpression of *GATA3-AS1* significantly enhanced SOD activity (Fig. 2F, p < 0.0001) and decreased MDA content (Fig. 2G, p < 0.001).

### The relationship between LncRNA GATA3-AS1 and miR-2116–5p

**Fig. 3:**
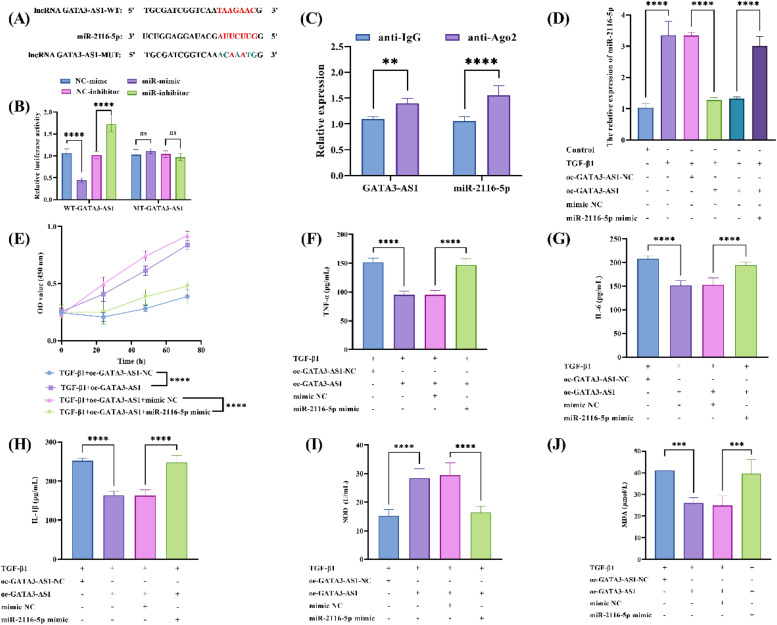
The interaction between *GATA3-AS1* and miR-2116–5p on the functional impact of TGF-β1-induced MPC5 cells. The binding sites sequence of *GATA3-AS1* with miR-2116–5p (A); Dual-luciferase reporter assay to validate the binding of *GATA3-AS1* with miR-2116–5p (B); RIP assay detects the enrichment of *GATA3-AS1* and miR-2116–5p (C); Relative expression levels of miR-2116–5p under different treatment condition (D); The cell proliferation capacity under different treatment conditions (E), TNF-α (F), IL-6 (G), IL-1β (H) levels, SOD activity (I), and MDA content (J); n = three biological replicates; Data were analyzed using one-way ANOVA for intergroup comparisons, followed by Tukey's post hoc test; ****p < 0.0001,***p < 0.001, **p < 0.01.

According to the analysis, in the WT sequence, *GATA3-AS1* and miR-2116–5p have complementary binding sites, whereas in the MUT sequence, this binding site is disrupted (Fig. 3A). The dual-luciferase reporter assay showed that, compared with NC-mimic, miR-2116–5p mimic significantly reduced the luciferase activity in the WT-*GATA3-AS1* group (p < 0.0001), while it had no significant effect on the MUT-*GATA3-AS1* group (Fig. 3B). Similarly, the miR-2116–5p inhibitor significantly increased the luciferase activity in the WT-*GATA3-AS1* group (p < 0.0001), while the NC-inhibitor had no significant effect. The RIP experimental results demonstrated that both *GATA3-AS1* (p < 0.01) and miR-2116–5p (p < 0.0001) were significantly enriched in the Ago2 immunoprecipitation complex compared to the IgG control group (Fig. 3C). Fig. 3D shows that TGF-β1 treatment significantly upregulates the expression of miR-2116–5p, and overexpression of *GATA3-AS1* reverses this effect, while miR-2116–5p mimic counteracts the regulatory role of *GATA3-AS1* (p < 0.0001). In addition, TGF-β1 inhibited cell proliferation, overexpression of *GATA3-AS1* promoted proliferation, but the miR-2116–5p mimic significantly attenuated the proliferative effect of *GATA3-AS1* (Fig. 3E, p < 0.0001). Overexpression of *GATA3-AS1* reduced the secretion of TNF-α (Fig. 3F), IL-6 (Fig. 3G), and IL-1β (Fig. 3H), while miR-2116–5p mimic reversed this inhibitory effect. Further analysis revealed that overexpression of *GATA3-AS1* enhanced SOD (Fig. 3I) and reduced MDA (Fig. 3J), while the miR-2116–5p mimic attenuated this protective effect.

### Prediction and functional analysis of miR-2116–5p target genes

**Fig. 4:**
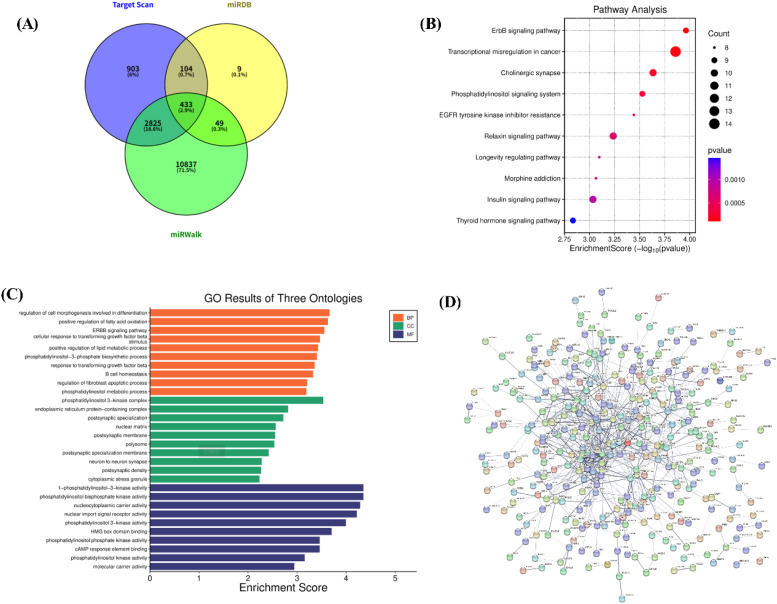
Prediction and Functional Analysis of miR-2116–5p Target Genes. Venn diagram of miR-2116–5p target gene prediction results (A), KEGG pathway enrichment analysis (B), GO functional enrichment analysis (C), and miRNA-target gene network analysis (D).

Using the miRmap, miRWalk, and TargetScan databases to predict potential target genes of miR-2116–5p, the Venn diagram shows 433 co-predicted genes, which may be high-confidence target genes of miR-2116–5p (Fig. 4A).

KEGG analysis showed these target genes were significantly enriched in signaling pathways closely related to inflammatory response, cell proliferation, and apoptosis (ErbB signaling pathway, Transcriptional misregulation in cancer, Cholinergic synapse, Phosphatidylinositol signaling system). miR-2116–5p may participate in the pathological process of childhood NS by regulating these pathways (Fig. 4B). GO enrichment analysis revealed (Fig. 4C) that in terms of biological processes, the target genes were significantly enriched in the regulation of cell morphogenesis, positive regulation of fatty acid oxidation, ERBB signaling pathway, and cellular response to transforming growth factor beta. These processes are closely related to the normal physiological functions of cells and the changes under pathological conditions, suggesting that miR-2116–5p may influence the progression of childhood NS by regulating these biological processes. The analysis of cellular components revealed that the target genes are primarily associated with cellular structures such as the phosphatidylinositol 3-kinase complex, the endoplasmic reticulum protein-containing complex, and the postsynaptic membrane. This indicates that the target genes of miR-2116–5p may play a significant role in cell signal transduction, protein synthesis and transport, as well as synaptic function. In terms of molecular functions, the target genes are significantly enriched in activities such as phosphatidylinositol bisphosphate kinase activity and nucleocytoplasmic transport signal receptor activity. These molecular functions are crucial for maintaining intracellular signal transduction, substance metabolism, and the stability of the cytoskeleton. In addition, the constructed miRNA-target gene network visually demonstrates the regulatory relationship between miR-2116–5p and its predicted target genes (Fig. 4D). Network analysis revealed the pivotal position of miR-2116–5p in the regulatory network and identified the top 10 core genes with the highest scores, including *TP53, TCF12, TAL1, SRC, SEMA3A, PRKDC*, PLXNA4, NRG4, NHEJ1, and JUN. The scores of both *TP53* and *TCF12* are 0.999, indicating their significant functional roles, and they are used for further research.

### The regulation of TCF12 by miR-2116–5p and its functional impact

**Fig. 5:**
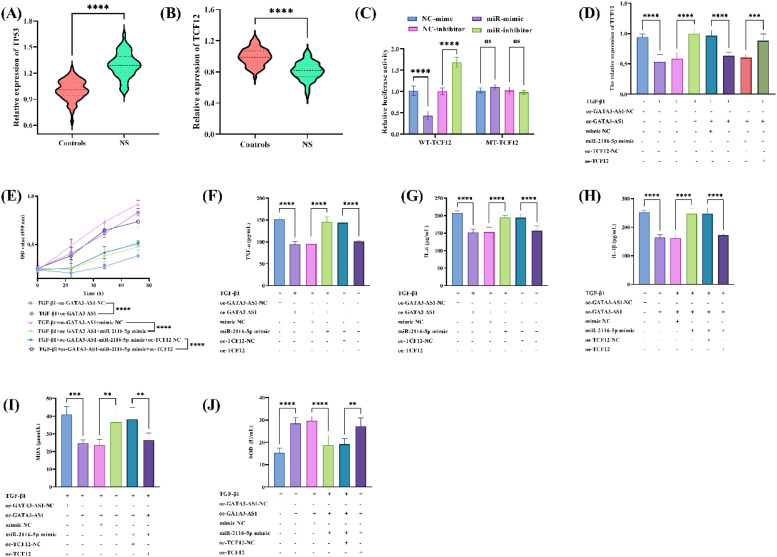
Functional analysis of the *GATA3-AS1*/miR-2116–5p/*TCF12* axis in childhood NS. The relative expression of *TP53* (A) and *TCF12* (B) in childhood NS patients and healthy controls; Luciferase activity validation of *TCF12* and miR-2116–5p (C); Relative expression of *TCF12* under different treatment conditions (D); Detection of cell proliferation capacity under different treatment conditions (E), TNF-α (F), IL-6 (G), IL-1β (H) levels, MDA content (I), and SOD activity (J); n = three biological replicates; Data were analyzed using one-way ANOVA for intergroup comparisons, followed by Tukey's post hoc test; ****p < 0.0001,***p < 0.001,**p < 0.01.

In childhood NS, the expression of *TP53* was significantly upregulated (Fig. 5A, p < 0.0001), while the relative level of *TCF12* was significantly lower than that in the healthy control group (Fig. 5B, p < 0.0001). The levels of *TP53* and miR-2116–5p were consistent, indicating that there was no targeted relationship between them. The dual-luciferase confirmed the binding of miR-2116–5p to *TCF12* (Fig. 5C). TGF-β1 induction significantly reduced *TCF12* expression levels (p < 0.0001). Overexpression of *GATA3-AS1* significantly restored *TCF12* levels (p < 0.0001), while the miR-2116–5p mimic further suppressed *TCF12* expression (p < 0.0001). Transfection with oe-*TCF12* reversed the *TCF12* inhibition mediated by the miR-2116–5p mimic (p < 0.001), further validating *TCF12* as a direct functional target of miR-2116–5p (Fig. 5D). Meanwhile, oe-*TCF12* rescued the *miR-2116-5p* mimic-induced suppression of proliferation (Fig. 5E), and significantly regulated intracellular inflammatory factors (p < 0.0001), including TNF-α (Fig. 5F), IL-6 (Fig. 5G), and IL-1β (Fig. 5H), as well as oxidative stress markers (p < 0.01), MDA (Fig. 5I) and SOD (Fig. 5J). Western Blot analysis showed that TGF-β1 treatment significantly reduced the expression of Synaptopodin and Podocin while upregulating Cleaved Caspase-3, whereas overexpression of *GATA3-AS1* effectively reversed this damage. However, co-transfection with the miR-2116–5p mimic partially counteracted the protective effect of *GATA3-AS1*. Under these conditions, further overexpression of *TCF12* was able to reverse the damage induced by the miR-2116–5p mimic, as reflected in the expression levels of Synaptopodin (Fig. S1A), Cleaved Caspase-3 (Fig. S1B), and Podocin (Fig. S1C).

### Knockdown of TCF12 reverses the protective effect of GATA3-AS1 on podocytes

To determine whether *TCF12* is essential in the *GATA3-AS1*-mediated protective pathway, we knocked down *TCF12* while overexpressing *GATA3-AS1*. The results showed that, compared with the control, knockdown of *TCF12* significantly reversed the protective effects of *GATA3-AS1* on podocytes. This reversal was manifested as decreased cell proliferation (Fig. S2A), increased secretion of inflammatory factors including TNF-α (Fig. S2B), IL-6 (Fig. S2C), and IL-1β (Fig. S2D), and aggravated oxidative stress reflected by changes in SOD (Fig. S2E) and MDA (Fig. S2F) levels. At the protein level, knockdown of *TCF12* also downregulated the expression of the podocyte cytoskeletal protein Synaptopodin (Fig. S2G) and the slit diaphragm protein Podocin (Fig. S2I), while reactivating the apoptosis marker protein Cleaved Caspase-3 (Fig. S2H).

## Discussion

The complexity of childhood NS stems from the interaction of various molecular and cellular mechanisms, as well as genetic and environmental factors, and its pathogenesis has not yet been fully elucidated. Previous studies have found that GATA3 expression is downregulated in renal cell carcinoma, and its overexpression can reduce tumor metastasis by inhibiting the STAT3 signaling pathway,^19^ suggesting that the dysregulation of *GATA3-AS1* is associated with renal pathology. We extended this research direction to pediatric NS, focusing on circulating molecules as noninvasive probes for disease pathology. Although renal biopsy is considered the gold standard, it is rarely performed in children with NS, especially in new-onset cases, due to ethical and clinical constraints. Therefore, this study utilized serum samples for analysis. Available evidence indicates that the levels of specific lncRNAs and miRNAs in serum have been shown to correlate with their expression in renal tissue and with the severity of kidney disease.^21^, ^22^, ^23^ This study is the first to identify a significant downregulation of LncRNA *GATA3-AS1* in the serum of pediatric NS patients, and the degree of this downregulation shows a negative correlation with key clinical indicators such as WBC, PLT, BUN, serum creatinine, and PRO.^24^, ^25^, ^26^ In summary, the systematic association of *GATA3-AS1* with key clinical indicators provides clues for its potential role as a circulating biomarker involved in the disease progression of childhood NS, and its clinical application value warrants further elucidation in future studies. In our cohort, LncRNA *GATA3-AS1* showed preliminary diagnostic efficacy for childhood NS (AUC = 0.892), which supports its potential for auxiliary diagnosis. Future studies with larger, longitudinal cohorts are warranted to validate the potential of serum *GATA3-AS1* and *miR-2116–5p* as a combined non-invasive biomarker panel for aiding diagnosis, monitoring disease activity, or assessing treatment response in childhood NS. Therefore, further investigation into the regulatory mechanisms of *GATA3-AS1* may provide new insights into the pathogenesis and diagnosis of childhood NS.

Podocytes are crucial for preserving the glomerular filtration barrier's integrity, their damage can lead to proteinuria and glomerulosclerosis.^27^ TGF-β1 plays a critical role in various kidney-related diseases and can exacerbate podocyte injury. For example, TGF-β1 can induce an increase in miR-155 expression in mouse podocytes, while simultaneously elevating the levels of pro-inflammatory cytokines.^28^ In addition, TGF-β1 can increase the expression of mitochondrial Nox4 by activating the mTORC1 pathway, leading to the generation of ROS and mitochondrial dysfunction, ultimately resulting in podocyte apoptosis.^29^ This study found that TGF-β1 treatment inhibited cell proliferation, upregulated inflammatory levels, and enhanced oxidative stress, while overexpression of *GATA3-AS1* significantly reversed these alterations. lncRNA can influence the processing, maturation, or stability of miRNA by directly binding to its precursor or mature form, thereby regulating miRNA function. This interaction can also be involved in the mechanisms of disease development and progression.^30^ The study found that lncRNA *GATA3-AS1* is upregulated in breast cancer tissues, and by binding to miR-495–3p, it indirectly regulates the expression of CENPU, thereby affecting the prognosis of breast cancer patients.^31^ The downregulation of *GATA3-AS1* can inhibit the proliferation of childhood ALL cells and induce cell apoptosis by targeting the upregulation of miR-515–5p expression.^32^ Based on this, we utilized the lncRNASNP2 database to predict the downstream miRNA of *GATA3-AS1* and selected miR-2116–5p with the highest binding score for further analysis. The luciferase activity assay confirmed the interaction between *GATA3-AS1* and miR-2116–5p Additionally, the Ago2 RIP experiment confirmed that *GATA3-AS1* binds with miR-2116–5p in the RISC complex, providing certain biochemical evidence for the ceRNA mechanism. Based on their genomic localization, it was found that *GATA3-AS1* (chromosome 10, https://www.ncbi.nlm.nih.gov/gene) and MIR2116 (chromosome 15, http://www.mirbase.org/) are located on different chromosomes. This analysis ruled out the possibility of both being regulated by a common upstream cis-regulatory element, further supporting the sponging mechanism. Further analysis revealed that miR-2116–5p is upregulated in childhood NS and significantly negatively correlated with *GATA3-AS1*, and possesses good diagnostic efficacy for childhood NS. In addition, miR-2116–5p can reverse the inhibitory effects of *GATA3-AS1* on inflammation and oxidative stress in TGF-β1-induced podocyte injury. It is speculated that miR-2116–5p may play a role in childhood NS by mediating *GATA3-AS1*.

Through the construction of a miRNA-mRNA regulatory network and screening of miR-2116–5p target genes, *TCF12* was identified as the research focus. Although previous studies have shown that *TCF12* acts as a transcriptional repressor of E-cadherin and promotes tumor metastasis in colorectal cancer,^33^ its role in kidney diseases remains unknown. This study found that *TCF12* expression is reduced in childhood NS. Overexpression of *TCF12* rescued miR-2116–5p-induced podocyte injury, while knockdown of *TCF12* reversed the protective effect of *GATA3-AS1*, demonstrating that *TCF12* is an indispensable downstream factor in this axis. Relevant studies have also reported that E-cadherin is a core molecule in EMT, and its downregulation is closely associated with pathologies such as renal fibrosis.^34^ Additional experiments further indicate that the *GATA3-AS1*/miR-2116–5p/*TCF12* axis significantly upregulates the expression of key podocyte cytoskeletal and slit‑diaphragm proteins, Synaptopodin and Podocin, and suppresses the activation of the apoptosis marker Cleaved Caspase-3. Therefore, this finding reveals a new function of *GATA3-AS1*/miR-2116–5p/*TCF12* axis in podocytes and provides a novel perspective for its investigation in kidney diseases.

This study offers fresh insights into the pathogenesis of childhood nephrotic syndrome and the screening of diagnostic markers, yet it is subject to several limitations. First, this study has a limited sample size, and all molecular tests were performed based on serum samples. Although changes in serum biomarkers are significantly correlated with disease activity, their expression and functional status in renal tissues, particularly in podocytes, still require direct confirmation. Future work will expand the cohort samples and utilize archived renal biopsy tissues or animal models to concurrently analyze the expression and correlation of this molecular axis in both serum and tissues. Second, the mechanism study relies entirely on a TGF-β1-induced mouse podocyte model, which, although capable of simulating key injury characteristics, cannot fully replicate the complex in vivo microenvironment and systemic disease processes. Future studies will validate the universality and functionality of this regulatory axis in models that more closely resemble human pathology, such as puromycin-induced nephropathy animal models. Third, the elucidation of the downstream transcriptional regulatory network of *TCF12* remains incomplete, with E-cadherin being only one of its potential targets. Future studies plan to systematically screen the gene sets regulated by *TCF12* through RNA-seq and validate its direct binding to key target gene promoters using ChIP-qPCR, thereby clarifying the specific mechanisms through which *TCF12* mediates podocyte protective effects via downstream molecules.

## Conclusion

This study found that *GATA3-AS1* is downregulated in childhood NS patients and negatively correlated with the expression of miR-2116–5p Overexpression of *GATA3-AS1* can restore TGF-β1-induced damage in MPC5 cells, while miR-2116–5p can reverse this protective effect. The study also revealed the direct binding relationship between miR-2116–5p and *TCF12*, indicating that *GATA3-AS1* influences podocyte function by regulating the miR-2116–5p-*TCF12* axis. These results provide a certain research background for subsequent studies on the roles of LncRNA *GATA3-AS1* and miR-2116–5p in childhood NS.

## Authors’ contributions

Conceptualization: X T, Y Z; Data curation: X T, P H; Formal analysis: X T, P H; Investigation: X T, P H; Methodology: X T, Y Z; Resources: Y Z; Software: Y Z; Writing-original draft: X T; Writing-review & editing: Y Z, P H;

## Funding

No funds, grants, or other support was received.

## Declaration of competing interest

All authors certify that they have no affiliations with or involvement in any organization or entity with any financial interest or non-financial interest in the subject matter or materials discussed in this manuscript.

## Data Availability

All data generated or analyzed during this study are included in this article. Further enquiries can be directed to the corresponding author.

## Appendix Supplementary materials

Image, application 1

## Footnotes

Supplementary material associated with this article can be found, in the online version, at doi:10.1016/j.clinsp.2026.101053.
