# Supplementary material for: LncRNA GATA3-AS1-mediated miR-2116-5p interaction modulates TCF12 and contributes to podocyte dysfunction in childhood nephrotic syndrome
Source: Clinics (Sao Paulo). 2026 Jul 16;81:101053. doi: 10.1016/j.clinsp.2026.101053 (PMC13383947; doi:10.1016/j.clinsp.2026.101053)

**CLINICS-D-25-01298_Supplementary Material**

**Figure S1** The roles of Synaptopodin, Cleaved Caspase-3, and Podocin in the *GATA3-AS1*/miR-2116-5p/*TCF12* axis. The relative protein expression levels of Synaptopodin (A), Cleaved Caspase-3 (B), and Podocin (C) after TGF-β1+oe-*GATA3-AS1*+miR-2116-5p mimic +oe-*TCF12* treatment; n = three biological replicates; Data were analyzed using one-way ANOVA for intergroup comparisons, followed by Tukey's post hoc test; ****p < 0.0001, ***p < 0.001, **p < 0.01, *p < 0.05.


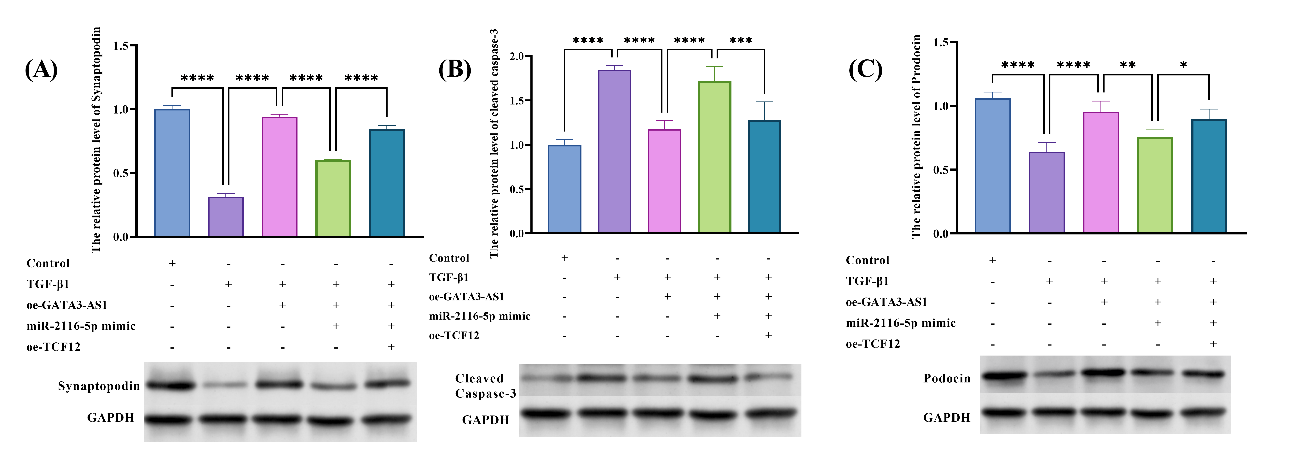


**Figure S2** Knockdown of *TCF12* reversed the effects of *GATA3-AS1* overexpression on cellular functions. Knockdown of *TCF12* reversed the effects of *GATA3-AS1* overexpression on cell proliferation (A), TNF-α (B), IL-6 (C), IL-1β (D), SOD (E), and MDA (F) levels; Knockdown of *TCF12* reversed the effects of *GATA3-AS1* overexpression on the expression levels of Synaptopodin (G), Cleaved Caspase-3 (H), and Podocin (I) proteins. n = three biological replicates; Data were analyzed using one-way ANOVA for intergroup comparisons, followed by Tukey's post hoc test; ****p < 0.0001.


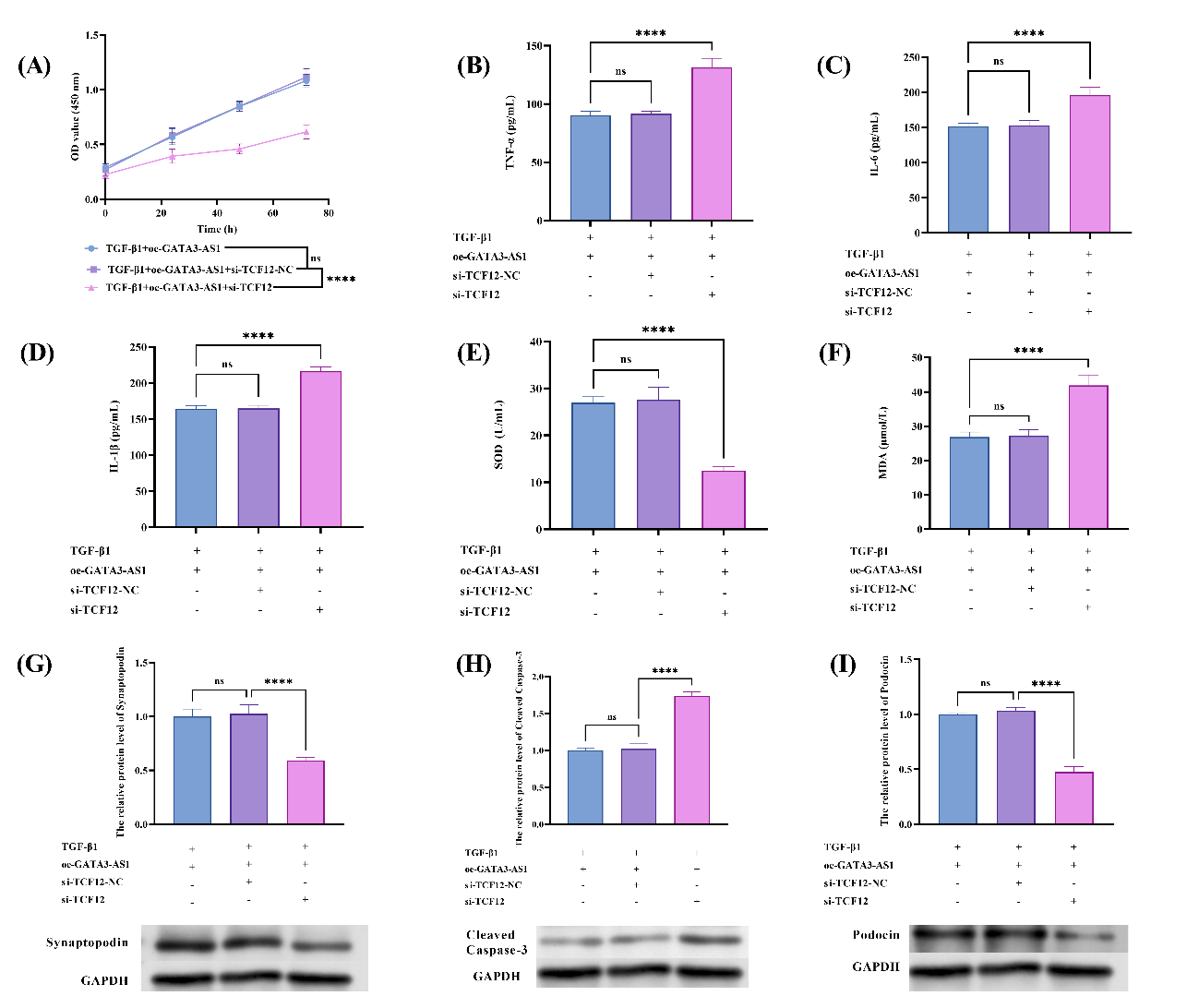

Supplement: Supplementary file 1 [file mmc1.docx]
